# Exploring the role of obesity in predicting mental health disorders: analyzing the effects of diagnosis sequence order

**DOI:** 10.1186/s12888-025-07427-6

**Published:** 2025-10-08

**Authors:** Kai-Jie Ma, Ming-Hsien Chou, Jen-De Chen, Ming-Qun Huang, Pei-Ying Tseng, Shu-Yuan Su, Jong-Yi Wang

**Affiliations:** 1 Department of Public Health, China Medical University, Taichung, 406040 Taiwan; 2 Department of Physical Medicine and Rehabilitation, Taichung Armed Forces General Hospital, Taichung, 411228 Taiwan; 3 Department of Sports, National Changhua University of Education, Changhua, 50007 Taiwan; 4 Department of Health Services Administration, China Medical University, Taichung, 406040 Taiwan; 5 Department of Medical, Lee’s General Hospital, Yuanli Town, Miaoli, 358011 Taiwan

**Keywords:** Obesity, Mental disorder, Diagnosis sequence, Two-way analysis

## Abstract

**Introduction:**

Mental disorders (MD) and obesity are significant global health concerns, with their interrelationship remaining unclear. While previous research has explored their association, the temporal sequence between these conditions has yet to be fully established. This study aims to investigate the bidirectional relationship between MD and obesity, identifying whether one condition precedes the other and assessing the risk associated with different MD subtypes.

**Methods:**

This retrospective cohort study utilized data from the Taiwan National Health Insurance Research Database. Patients diagnosed with MD or obesity were included, with those having prior diagnoses of both conditions excluded. A two-year washout period was applied to confirm disease onset sequence. Propensity score matching was conducted at a 1:1 ratio based on gender, age, Charlson Comorbidity Index, urbanization level, and insurance amount. A total of 171,056 participants were included in the final analysis. Cox proportional hazards models were used to assess the risk of developing MD after obesity and vice versa.

**Results:**

Survival analysis revealed no significant association between obesity and the subsequent development of MD. However, patients with MD had a significantly increased risk of developing obesity (aHR = 1.11, *p* = 0.03). Among MD subtypes, schizophrenia was associated with the highest risk of obesity (aOR = 2.05, *p* < 0.01), followed by affective disorders (aOR = 1.42, *p* = 0.01) and anxiety disorders (aOR = 1.35, *p* = 0.01).

**Conclusion:**

This study suggests that MD, particularly schizophrenia, is associated with an increased risk of obesity, whereas obesity does not appear to significantly elevate the risk of MD, indicating a unidirectional association. The findings highlight the need for proactive weight management strategies in MD patients to mitigate obesity-related health risks.

**Supplementary Information:**

The online version contains supplementary material available at 10.1186/s12888-025-07427-6.

## Introduction

Mental disorder (MD) is an increasing global health issue with rising numbers of people being diagnosed with the condition. According to the 2019 WHO’s statistics, there were approximately 1 billion people with MD [1]. The national prevalence of MD increased from 8.89% in 2008 to 11.98% in 2019 [2]. Not only does MD increase the financial burden on national medical service facilities, but it also impacts the economy [3–5]. Furthermore, MD not only causes functional mental damage but is often accompanied by more than one chronic disease [6, 7] and obesity [8, 9].

Obesity has become a global problem [10] caused by changes in modern life and concurrent stress [11]. Statistics from the World Obesity Federation show that more than 1 billion people have concerns about obesity. Obesity, or an overweight condition, affects more than 60% of the adult population, and about 1/3 of the children in Europe are overweight. This not only reduces life expectancy [12, 13] but also has an impact on national economies [14–17]. Obesity can result from MD due to the disease status and medication [13, 18, 19]. Studies have also revealed that social perception of obesity can have a psychosomatic effect on obese patients, resulting in MD [20–22]. This is the reason why the order of correlation has always been a debatable issue.

Past research has mainly focused on the connection between obesity and MD. However, no definitive conclusion has been reached about the correlation between these two conditions. Few studies have been made that explore the order of correlation, and attention should be given to the health of the obese population, which continues to increase in Taiwan [23]. The aim of this study was to gain a better understanding of the order of correlation between obesity and MD. To do this, the participants were divided into two comparable groups. In one group, the relevant risks of obese patients developing MD were investigated and in the other group, the relevant risks of MD patients becoming obese were studied. This is the first study focused on an in-depth investigation of the correlation between obesity and MD and the various types of MD involved. The design of this study is somewhat unusual. In the past there had yet been studies simultaneously researching over the two types of time correlation, but we expect the results to be of considerable use in preventative health care. They should also raise awareness of self-protection, lower the rate of disease, and reduce medical expenditure and the cost of health services.

Therefore, the main objective of this study is to explore the bidirectional relationship between obesity and MD, aiming to understand the sequence of these health conditions. The research questions are whether obesity increases the risk of developing MD and whether MD increases the risk of subsequently developing obesity, along with identifying which specific type of MD is involved. Finally, the research hypotheses are that patients with obesity have a higher risk of developing MD compared to patients with without obesity, and that patients with MD have a higher risk of developing obesity compared to those without MD.

## Methods

### Study design

This study adopted a retrospective cohort design to explore the relationship between obesity and mental disorders. We utilized two study branches for the analysis. First, for patients who developed obesity prior to developing a mental disorder, we established a two-year observation period to ensure the temporal sequence of disease onset. Eligible patients were required to have no mental disorder diagnosis in the two years preceding their initial diagnosis of obesity. Subsequently, patients without obesity were selected as the control group, and propensity score matching (PSM) was performed based on gender, age, Charlson Comorbidity Index, urbanization level, and insurance amount, with a matching ratio of 1:1. Second, for patients who developed a mental disorder prior to developing obesity, we similarly established a two-year observation period to ensure the sequence of the two diseases and selected patients without mental disorders as the control group, using the same matching criteria for 1:1 PSM.

### Data sources

This retrospective study used a secondary database. The study period was from 2002 to 2013, and the data were collected from 1 million random records provided by the National Health Insurance Research Database (NHIRD). Our study was approved by the Research Ethics Committee of China Medical University Hospital, Taiwan. To protect the patients’ privacy, all personal identification numbers were en-crypted by the National Health Research Institutes before the data were released. The Taiwan National Health Research Institutes encrypts patients’ personal information to protect privacy and provides researchers with anonymous scrambled identification numbers associated with relevant disease information. Therefore, a patient informed con-sent is not required for authorized researchers to access this research database.

### National health insurance research database

The NHIRD was first established in 1995 and is a national-level health database currently managed by the Ministry of Health and Welfare. It is the most comprehensive electronic health record in Taiwan, and all applications and uses are subject to strict review by relevant authorities. The NHIRD covers more than 99.6% of Taiwan’s population, including demographic variables, outpatient and inpatient information, prescriptions, diagnostic information, healthcare provider details, and other comprehensive clinical information. Any healthcare behaviors of the public are recorded. The database is divided into full data files and sampled data files. The sampling method is stratified by gender, age, and region, with the sample size for each stratum calculated based on its proportion in the overall population. The sampling method used is random sampling. This study utilized the sampled dataset.

### Charlson comorbidities index

Our research used the Charlson Comorbidity Index (CCI) developed by Charlson in 1984, to evaluate the mortality risk and burden of disease, address the confounding influence of comorbidities, and predict outcomes. We followed the method proposed by Charlson; CCI consists of 17 comorbidities, weighted from 1 to 6 according to mortality risk and disease severity, and then summed scores to form the total CCI score [24]. However, subjects rarely displayed high CCI scores in our research, so we divided the CCI categories into two groups as follows: 0 points and more than 1 points.

### Inclusion and exclusion criteria

Our study included patients identified as having mental disorders and obesity based on the International Classification of Diseases, Ninth Revision, Clinical Modification (ICD-9-CM). Mental disorders were categorized into five types: affective disorder, anxiety disorder, substance use disorder, schizophrenia, and other MD, according to the Diagnostic and Statistical Manual of Mental Disorders, Fifth Edition (DSM-5). Additionally, patients identified as having obesity were those with an ICD-9-CM code of 278, while patients without this code were defined as non-obese. The first of the two groups in this study was comprised of 15,688 patients who had been diagnosed with obesity and had been included in the ICD-9-CM. A total of 1,508 underage patients were excluded. To confirm that no MD had been observed before diagnosis. A two-year observation period resulted in the elimination of 4,801 subjects. A one-by-one propensity score matching (PSM) was then carried out on this group based on gender, age, CCI, degree of urbanization, and income. A total of 9,900 patients remained (Table S1 & S3). There were 285,127 patients in the second group diagnosed with MD using ICD-9-CM and 22,276 underage individuals were excluded. To confirm that no obesity had been observed before diagnosis, a two-year observation period was set, and 3.553 subjects were excluded. Then one by one PSM was carried out based on gender, age, CCI, and degree of urbanization, and 171,056 subjects remained (Table S2 & S4).

### Statistical analysis

The one by one PSM was based on gender, age, CCI, degree of urbanization, and income after the study sample had been screened under specific inclusion and exclusion criteria. Descriptions were given to the samples to elucidate the distribution of characteristics within the two study groups. Then Pearson’s chi-squared test was used to judge the features and the correlation between MD and obesity. Collinearity diagnostics were conducted prior to the regression analysis to ensure that no adverse effects on the primary influencing factors (main independent variables and control variables) led to biased results. Collinearity diagnostics typically use the Variance Inflation Factor (VIF) to determine the presence of collinearity, with values greater than 10 indicating potential issues. In this study, all VIF values were below 10. For regression analysis, we first used Cox regression to determine the risk between obesity and MD, as Cox regression allows for the inclusion of time between diseases in the analysis. Subsequently, MD was further classified into five types according to the DSM-5 and its corresponding ICD-9-CM codes, and multiple logistic regression was used to investigate which type of MD obese patients were more prone to develop. Additionally, when exploring the relationship between obesity and MD, we included potential influencing factors identified in previous studies. These included demographic variables (age, sex, occupation [25–28], urbanization level [29, 30], and income), clinical factors (CCI), and medication use [31, 32], specifically drugs known to have obesity-related side effects. Medication use was included as a covariate in the regression models to control for its potential confounding effect, ensuring that our findings were not biased by the impact of pharmacological weight gain. Data analysis was done using SAS 9.4.

## Results

Analyses were carried out with 9,900 subjects who were first diagnosed with obesity and then with MD and 171,056 subjects who were first diagnosed with MD and then with obesity. There were 28.85% positive MD diagnoses in the obesity before MD group, and the majority (1,423 subjects) had anxiety disorders. In the other group (MD before obesity), 2,516 subjects (46.18%) had anxiety disorders, see Table 1. Chi-Squared Tests on the MD type and obesity (Table S5, S6) showed a significance of *P* < 0.01. The Collinearity Diagnostics that were carried out at the same time showed an independent covariate of VIF < 10.

**Table 1:** Characteristics of the samples studied

Variable	First diagnosed with obesity and then with MD		First diagnosed with MD and then with obesity	
	*N*	%	*N*	%
Obesity				
present	4,950	50.00	168,540	98.53
absent	4,950	50.00	2,516	1.47
MD				
present	7,044	71.15	85,528	50.00
absent	2,856	28.85	85,528	50.00
MD-type				
absent	7,044	71.15	85,528	50.00
affective disorder	221	2.23	8,196	9.58
anxiety disorder	1,423	14.37	39,498	46.18
substance use disorder	219	2.21	8,270	9.67
schizophrenia	42	0.42	1,406	1.64
others MD	951	9.61	28,158	32.92
Gender				
female	5,604	56.61	85,065	49.73
male	4,296	43.39	85,991	50.27
Age (years)				
20–24	836	8.44	6,551	3.83
25–34	1,766	17.84	25,194	14.73
35–44	2,422	24.46	34,902	20.40
45–54	2,034	20.55	36,033	21.07
55–64	1,758	17.76	31,696	18.53
>=65	1,084	10.95	36,680	21.44
Comorbidity (CCI)				
0	8,386	84.71	125,838	73.57
>=1	1,514	15.29	45,218	26.43
Income (NTD)				
<=20,008	6,364	64.28	118,088	69.03
20,009–22,800	1,172	11.84	15,741	9.20
22,801–28,800	546	5.52	9,046	5.29
28,801–36,300	620	6.26	8,882	5.19
36,301–45,800	638	6.44	9,599	5.61
45,801–57,800	198	2.00	3,852	2.25
57,801–72,800	156	1.58	3,102	1.81
>=72,801	206	2.08	2,746	1.61
Occupation				
unemployed	1,414	14.28	16,965	9.92
private employee and government	5,035	50.86	77,486	45.30
labor union member	1,234	12.46	23,801	13.91
farmer and fisherman	412	4.16	18,089	10.57
soldier	27	0.27	0	0.00
social	130	1.31	2,013	1.18
veteran	1,648	16.65	32,702	19.12
Level of urbanization				
highly urbanized area	3,486	35.21	52,852	30.90
moderately urbanized area	3,256	32.89	51,658	30.20
emerging area	1,588	16.04	28,014	16.38
general area	1,080	10.91	23,106	13.51
aging area	132	1.33	4,100	2.40
agricultural area	180	1.82	6,033	3.53
remote area	178	1.80	5,293	3.09
Location				
taipei division	4,194	42.36	63,160	36.92
northern division	1,570	15.86	24,840	14.52
central division	1,555	15.71	30,643	17.91
southern division	1,045	10.56	22,526	13.17
kaoping division	1,298	13.11	25,884	15.13
eastern division	238	2.40	4,003	2.34
Taking drugs with obesity side effects				
no	-	-	170,021	99.39
yes	-	-	1,035	0.61

### The correlation between obesity and MD

Adjusted Cox regression analyses clearly showed no significant correlation between obesity and MD in patients with obesity who developed MD later, see Table 2. There were no other significant correlations between occupation, residential area, etc., and MD acquisition. However, the cox regression analyses for the group where MD was acquired before obesity (Table 3) indicated that individuals with MD had a greater risk of subsequently developing obesity compared to the control group without MD (aHR = 1.11, *p* = 0.03). This aHR of 1.11 suggests that, after controlling for covariates, individuals with MD had a 11% higher risk of acquiring obesity compared to those without MD, and this association was statistically significant (*p* < 0.05). Compared to those unemployed, the risk of obesity was lower in private employees, government employees, union members, and veterans (aHR = 0.79–0.84, *p* < 0.05). However, an unexpected result was that those using drugs with the side-effect of obesity had a lower risk of obesity (aHR = 0.72, *p* = 0.04). There was no significant correlation between residential area and the development of obesity.

**Table 2:**
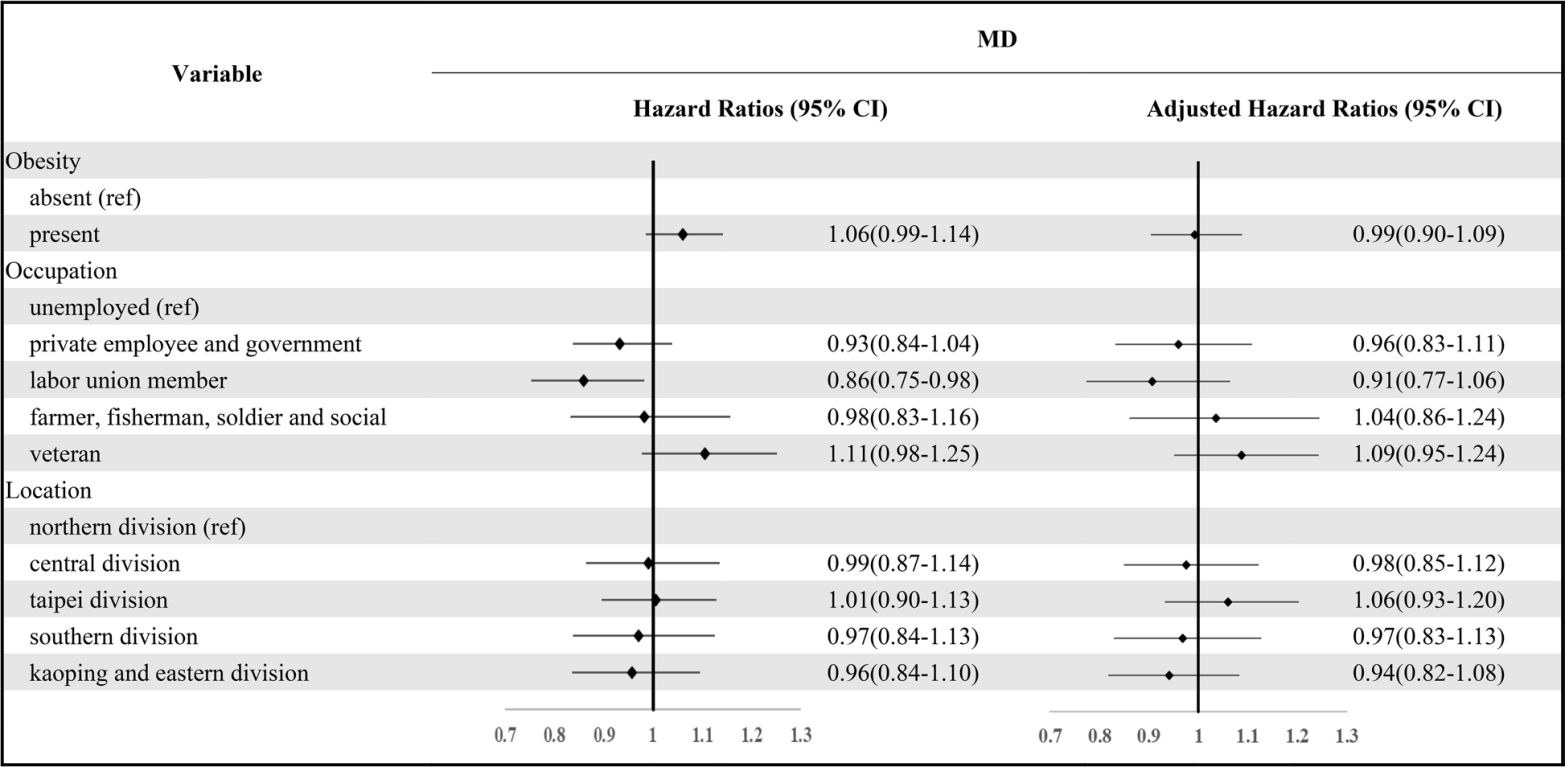
Risk of developing MD in patients with obesity

The Adjusted Hazard Ratio was estimated after controlling for covariate variables such as occupation and location

**Table 3:**
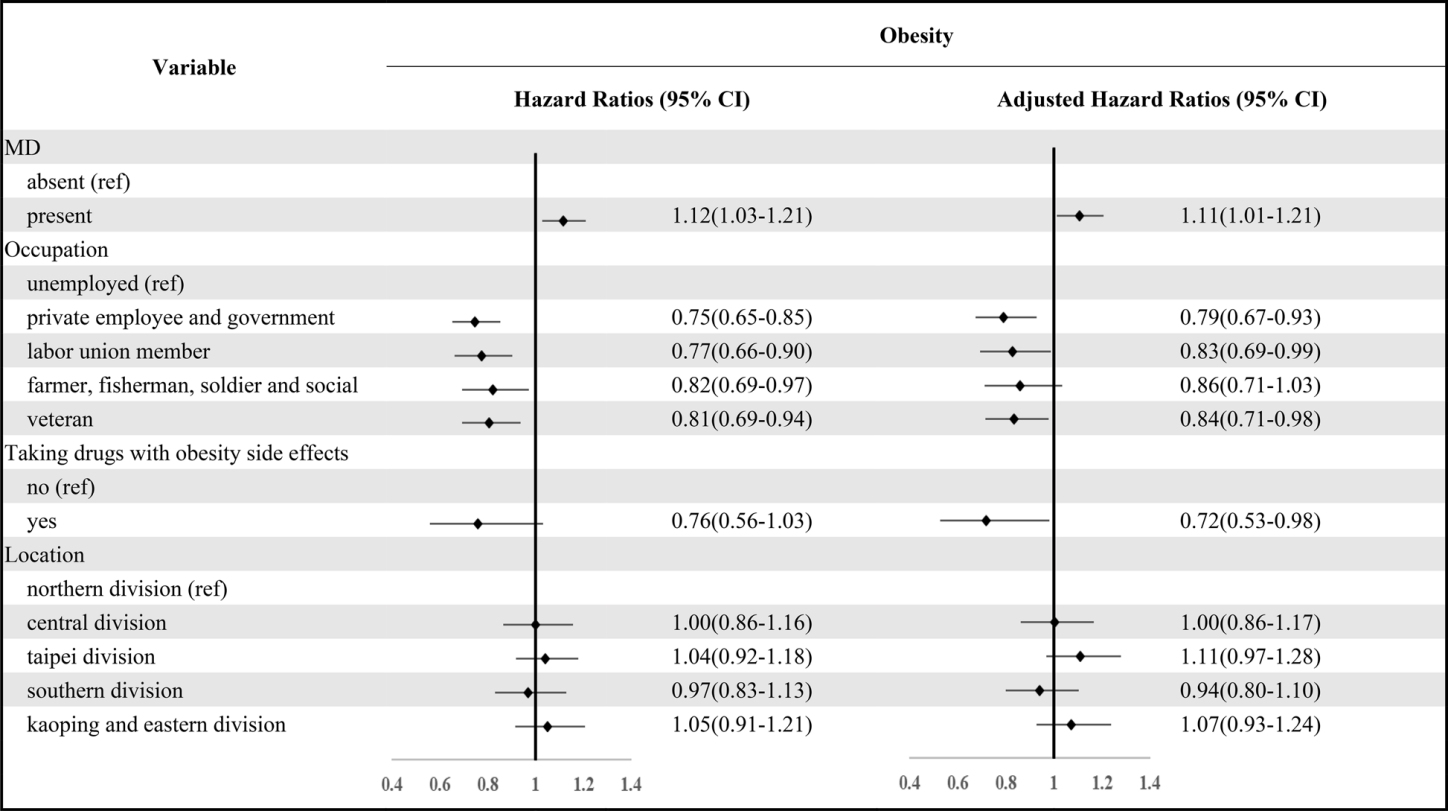
Obesity risk within three years among patients diagnosed with mental disorders

The Adjusted Hazard Ratio was estimated after controlling for covariate variables such as occupation, taking drugs with obesity side effects and location

### Risk for MD types

Cox regression analyses showed that the group with MD before obesity was significant, and an effort was made to discover the type of MD that would present the greatest risk for obesity. The regression model showed that subjects with schizophrenia had the most significant risk for obesity (see Table 4); the risk was 2.05 times greater (aOR = 2.05, *P* < 0.01) than in the control group. The second most prevalent risk was for affective disorder (aOR = 1.42, *P* = 0.01), the third anxiety disorder (aOR = 1.35, *P* = 0.01), and the fourth was other disorders MDs (aOR = 1.28, *P* = 0.04). It was also found that if a subject had acquired more than two types of MD, the risk of obesity also increased (aOR = 1.39, *P* < 0.01). Another interesting aspect was that the use of drugs with a side effect of obesity lowered the risk of obesity (Table 3). A revert display of the regression model revealed that those using drugs with obesity side effects had increased risk of obesity (aOR = 1.75, *P* < 0.01). There was no significant correlation between residential area and occupation.

**Table 4:**
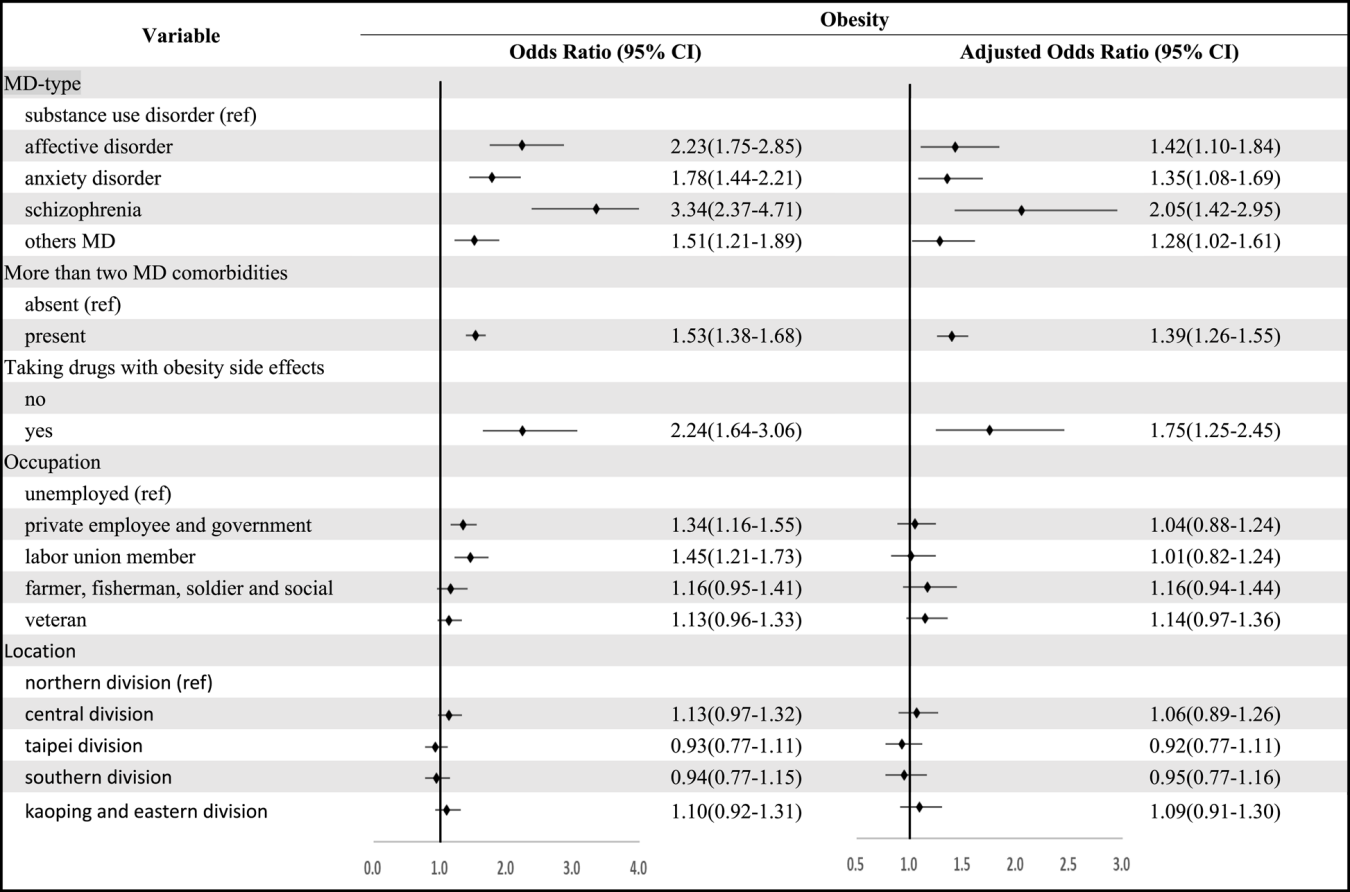
Association between mental disorders and later obesity risk

The Adjusted Odds Ratio was estimated after controlling for covariate variables such as more than two MD comorbidities, taking drugs with obesity side effects, occupation and location

## Discussion

The aim of this study was to investigate possible correlation between obesity and MD in two selected groups. Past studies have shown that obese patients have low self-esteem and cannot meet the social aesthetics standards because they believe they are attractive or lack charm. This places them under an even heavier psychological burden and further harms their mental health, leading to more MD [33, 34]. In addition, some earlier studies suggest that depression is a cause of obesity as a health consequence that could originate in childhood; it could have a genetic, familial, societal, or even environmental background [35].

A correlation was discovered in the MD before obesity group using the Cox regression or regression models. Earlier studies show that patients with depression may be more likely to become obese due to metabolic disturbances caused by a lack of exercise or an irregular diet [36]. Furthermore, some studies revealed that depressed patients tend to have particular dietary preferences, such as a preference for sweet or high-calorie foods, which may contribute to an increased risk of developing obesity [37, 38]. In addition to inadequate exercise and eating habits, it has also been shown that long-term use of antidepressants and endocrine disorders have been suggested to be associated with weight gain in patients with depression [34]. Alcohol abuse in patients with anxiety disorder could also result in excess calorie intake that leads to weight gain [39]. It has also been suggested that neuropeptide Y (NPY), which serves as a medium for pressure exposure in adipose tissue, leads to weight gain in patients with anxiety disorder. High sugar and fat intake that leads to weight gain could result from increased NPY conduction from glucocorticoids at high-stress levels [40]. Some past studies have also shown schizophrenia can be caused by the side effects of atypical antipsychotics. Although the mechanism by which these drugs cause weight loss requires further clarification, it was apparent that the effect depended on the type of atypical antipsychotics being used. In particular, the weight gain caused by olanzapine and clozapine was most obvious [19].

That patients with more than two types of MD had a greater risk of obesity was not surprising because it is known that this might be caused by multiple MD drug use. Lanzapine and clozapine, risperidone, and ziprasidone are known to have obesity as a side effect, and weight gains can be seen in those receiving treatment over 10 weeks [19]. A large Pan-American study carried out in more than 50 different locations revealed [41] that second-generation antipsychotics could affect the metabolic syndrome, resulting in obesity. More than 41% of patients with MD had metabolic syndrome. It was also discovered that other MD medications, such as mood stabilizers, could cause obesity [42]. It is important to note, however, that these conclusions regarding the role of specific medications in causing obesity are primarily drawn from previous literature rather than solely from our study’s statistical analysis.

In addition, recent evidence has highlighted the complex interplay between obesity and psychological outcomes. For instance, a study examined women with fibromyalgia and comorbid obesity, finding that depressive symptomatology, sleep quality, and pain catastrophizing were independently associated with suicidal ideation [43]. This underscores that obesity can exacerbate mental health vulnerabilities, supporting the importance of monitoring obesity-related risk factors in individuals with mental disorders.

This study did not reveal any correlation between obesity with occupation and residential area. However, as in many previous studies, veterans were found to be the MD group with the highest risk [44–46]. Furthermore, the risk level for this category was the same for all types of MD. It was found that the Southern, Kaoping, and Eastern Division residential areas had high risk for MD. Past studies suggest that low job opportunities and unfavorable living conditions cause an increase in the risk of acquiring MD [47]. Interestingly, our study found that patients using obesity-inducing medications had a lower risk of developing obesity, which contrasts with findings from previous research. A possible explanation is that these MD patients receive more intensive medical monitoring, leading to earlier intervention and weight management. Additionally, the continuity of care for MD patients in Taiwan is relatively high, allowing physicians to frequently adjust medications to mitigate excessive weight gain.

Our study has several limitations. First, we used secondary data, which inherently has certain constraints. Second, the diagnostic criteria for identifying mental disorders and obesity were based solely on ICD-9-CM, and the database did not include treatment details, which limited our ability to use specific care and standards for further identification of mental disorders. Additionally, the database lacked data on height and weight, which may have led to the omission of patients with potential obesity issues. Third, the use of the CCI may have introduced bias, as it does not fully capture the specific characteristics of mental disorders, limiting the accuracy of our findings related to mental health. These limitations may have influenced our study results. Fourth, due to data access restrictions, we were unable to exclude patients taking medications known to induce obesity. Although this could introduce potential confounding, we addressed this limitation by incorporating medication use as a covariate in all adjusted models.

Given the limitations identified in this study, future research should consider two key improvements. First, utilizing databases that include comprehensive mental health assessments, detailed clinical diagnoses, and treatment information could help address the limitations of using the CCI, particularly in capturing the specific characteristics of mental disorders, and allow for a more precise evaluation of mental health and obesity. Second, adopting databases with detailed clinical diagnoses and treatment information would enable more accurate categorization of mental disorders and obesity. These improvements would enhance the precision of study findings, especially in examining the complex relationship between these conditions.

## Conclusion

The relationship between obesity and mental health problems has been widely debated. The results of this study provide further insight into this issue. Our findings clarify the association between affective disorders, anxiety disorders, schizophrenia, and other mental disorders with obesity. While a strong association was observed, our study design does not establish a causal link. Future research using longitudinal and interventional approaches is needed to further explore potential causal mechanisms.

## Supplementary Information

Supplementary Material 1.

## Data Availability

The data that support the findings of this study are available from the Health and Welfare Data Science Center, but restrictions apply to the availability of these data, which were used under license for the current study, and so are not publicly available. Data are however available from the corresponding author (Jong-Yi Wang, E-mail: Jong-Yi Wang) upon reasonable request and with permission of Health and Welfare Data Science Center.

## Abbreviations

MD: Mental Disorders
aHR: Adjusted Hazard Ratio
aOR: Adjusted Odds Ratio
PSM: Propensity Score Matching
CCI: Charlson Comorbidity Index
NHIRD: National Health Insurance Research Database
VIF: Variance Inflation Factor
DSM-5: Diagnostic and Statistical Manual of Mental Disorders, Fifth Edition
NPY: Neuropeptide Y
MOHW: Health and Welfare
