# Supplementary material for: Exploring the role of obesity in predicting mental health disorders: analyzing the effects of diagnosis sequence order
Source: BMC Psychiatry. 2025 Oct 8;25:946. doi: 10.1186/s12888-025-07427-6 (PMC12506265; doi:10.1186/s12888-025-07427-6)
Supplement: Supplementary file 1 — Supplementary Material 1. [file 12888_2025_7427_MOESM1_ESM.docx]

PSM at a 1:1 ratio was conducted based on gender, age, CCI, urbanization level, and insurance amount.

Patients without obesity
(N=982,509)

Random sampling data file of the NHIRD
(N=1,000,000)

ICD-9-CM:278

Patients with obesity
(N=17,491)

Exclude patients diagnosed with obesity during the observation period
(N=15,688)

Exclude individuals under the age of 20
(N=14,180)

Exclude patients diagnosed with mental disorders during the observation period
(N=9,379)

Patients with obesity
(N=4,950)

Patients without obesity
(N=4,950)

Table S1. Flowchart of sample selection for patients with obesity

PSM at a 1:1 ratio was conducted based on gender, age, CCI, urbanization level, and insurance amount.

Patients without mental disorder
(N=640,870)

Random sampling data file of the NHIRD
(N=1,000,000)

ICD-9-CM:290-319

Patients with mental disorder
(N=359,130)

Exclude patients diagnosed with mental disorders during the observation period
(N=285,127)

Exclude individuals under the age of 20
(N=262,851)

Exclude patients diagnosed with obesity during the observation period
(N=259,298)

Patients with mental disorder
(N=85,528)

Patients without mental disorder
(N=85,528)

Table S2. Flowchart of sample selection for patients with mental disorder

Table S3. Chi-square test results for matched covariates in patients with and without obesity

| **Variable** | **Patients with obesity** | | **Patients without obesity** | | **P-value** |
| --- | --- | --- | --- | --- | --- |
|  | **N** | **%** | **N** | **%** |  |
| Gender |  |  |  |  | 1.0000 |
| female | 2,148 | 43.39 | 2,148 | 43.39 |  |
| male | 2,802 | 56.61 | 2,802 | 56.61 |  |
| Age (years) |  |  |  |  | 1.0000 |
| 20-24 | 418 | 8.44 | 418 | 8.44 |  |
| 25-34 | 883 | 17.84 | 883 | 17.84 |  |
| 35-44 | 1,211 | 24.46 | 1,211 | 24.46 |  |
| 45-54 | 1,017 | 20.55 | 1,017 | 20.55 |  |
| 55-64 | 879 | 17.76 | 879 | 17.76 |  |
| >=65 | 542 | 10.95 | 542 | 10.95 |  |
| Comorbidity (CCI) |  |  |  |  | 1.0000 |
| 0 | 4,193 | 84.71 | 4,193 | 84.71 |  |
| 1 | 713 | 14.40 | 713 | 14.40 |  |
| >=2 | 44 | 0.89 | 44 | 0.89 |  |
| Level of urbanization |  |  |  |  | 1.0000 |
| highly urbanized area | 1,743 | 35.21 | 1,743 | 35.21 |  |
| moderately urbanized area | 1,628 | 32.89 | 1,628 | 32.89 |  |
| emerging area | 794 | 16.04 | 794 | 16.04 |  |
| general area | 540 | 10.91 | 540 | 10.91 |  |
| aging area | 66 | 1.33 | 66 | 1.33 |  |
| agricultural area | 90 | 1.82 | 90 | 1.82 |  |
| remote area | 89 | 1.80 | 89 | 1.80 |  |
| Income (NTD) |  |  |  |  |  |
| <=20008 | 3,182 | 64.28 | 3,182 | 64.28 | 1.0000 |
| 20009-22800 | 586 | 11.84 | 586 | 11.84 |  |
| 22801-28800 | 273 | 5.52 | 273 | 5.52 |  |
| 28801-36300 | 310 | 6.26 | 310 | 6.26 |  |
| 36301-45800 | 319 | 6.44 | 319 | 6.44 |  |
| 45801-57800 | 99 | 2.00 | 99 | 2.00 |  |
| 57801-72800 | 78 | 1.58 | 78 | 1.58 |  |
| >=72801 | 103 | 2.08 | 103 | 2.08 | 1.0000 |

Table S4. Chi-square test results for matched covariates in patients with and without mental disorder

| **Variable** | **Patients with mental disorder** | | **Patients without mental disorder** | | **P-value** |
| --- | --- | --- | --- | --- | --- |
|  | **N** | **%** | **N** | **%** |  |
| Gender |  |  |  |  | 0.9961 |
| female | 42,996 | 50.27 | 42,995 | 50.27 |  |
| male | 42,532 | 49.73 | 42,533 | 49.73 |  |
| Age (years) |  |  |  |  | 1.0000 |
| 20-24 | 3,277 | 3.83 | 3,274 | 3.83 |  |
| 25-34 | 12,598 | 14.73 | 12,596 | 14.73 |  |
| 35-44 | 17,452 | 20.41 | 17,450 | 20.40 |  |
| 45-54 | 18,016 | 21.06 | 18,017 | 21.07 |  |
| 55-64 | 15,847 | 18.53 | 15,849 | 18.53 |  |
| >=65 | 18,338 | 21.44 | 18,342 | 21.45 |  |
| Comorbidity (CCI) |  |  |  |  | 0.9999 |
| 0 | 62,918 | 73.56 | 62,920 | 73.57 |  |
| 1 | 19,633 | 22.96 | 19,631 | 22.95 |  |
| >=2 | 2,977 | 3.48 | 2,977 | 3.48 |  |
| Level of urbanization |  |  |  |  | 1.0000 |
| highly urbanized area | 26,429 | 30.90 | 26,423 | 30.89 |  |
| moderately urbanized area | 25,829 | 30.20 | 25,829 | 30.20 |  |
| emerging area | 14,006 | 16.38 | 14,008 | 16.38 |  |
| general area | 11,552 | 13.51 | 11,554 | 13.51 |  |
| aging area | 2,050 | 2.40 | 2,050 | 2.40 |  |
| agricultural area | 3,016 | 3.53 | 3,017 | 3.53 |  |
| remote area | 2,646 | 3.09 | 2,647 | 3.09 |  |
| Income (NTD) |  |  |  |  |  |
| <=20008 | 59,045 | 69.04 | 59,043 | 69.03 | 1.0000 |
| 20009-22800 | 7,872 | 9.20 | 7,869 | 9.20 |  |
| 22801-28800 | 4,523 | 5.29 | 4,523 | 5.29 |  |
| 28801-36300 | 4,442 | 5.19 | 4,440 | 5.19 |  |
| 36301-45800 | 4,798 | 5.61 | 4,801 | 5.61 |  |
| 45801-57800 | 1,925 | 2.25 | 1,927 | 2.25 |  |
| 57801-72800 | 1,550 | 1.81 | 1,552 | 1.81 |  |
| >=72801 | 1,373 | 1.61 | 1,373 | 1.61 |  |

| Table S5. Chi-square test of matched patient samples for MD | | | | |  |  |
| --- | --- | --- | --- | --- | --- | --- |
| **Variable** | **MD** | | | | | **P-value** |
|  | **absent** | |  | **present** | |  |
|  | **N** | **%** |  | **N** | **%** |  |
| **Obesity** |  |  |  |  |  | <.01** |
| absent | 3,623 | 73.19 |  | 1,327 | 26.81 |  |
| present | 3,421 | 69.11 |  | 1,529 | 30.89 |  |
| **Comorbidity (CCI)** |  |  |  |  |  | <.01** |
| 0 | 6,697 | 79.86 |  | 1,689 | 20.14 |  |
| >=1 | 347 | 22.92 |  | 1,167 | 77.08 |  |
| Gender |  |  |  |  |  | <.01** |
| female | 3,784 | 67.52 |  | 1,820 | 32.48 |  |
| male | 3,260 | 75.88 |  | 1,036 | 24.12 |  |
| Age (years) |  |  |  |  |  | <.01** |
| 20-24 | 706 | 84.45 |  | 130 | 15.55 |  |
| 25-34 | 1,427 | 80.8 |  | 339 | 19.2 |  |
| 35-44 | 1,834 | 75.72 |  | 588 | 24.28 |  |
| 45-54 | 1,435 | 70.55 |  | 599 | 29.45 |  |
| 55-64 | 1,121 | 63.77 |  | 637 | 36.23 |  |
| >=65 | 521 | 48.06 |  | 563 | 51.94 |  |
| Income (NTD) |  |  |  |  |  | <.01** |
| <=20008 | 4,379 | 68.81 |  | 1,985 | 31.19 |  |
| 20009-22800 | 868 | 74.06 |  | 304 | 25.94 |  |
| 22801-28800 | 416 | 76.19 |  | 130 | 23.81 |  |
| 28801-36300 | 476 | 76.77 |  | 144 | 23.23 |  |
| 36301-45800 | 474 | 74.29 |  | 164 | 25.71 |  |
| 45801-57800 | 151 | 76.26 |  | 47 | 23.74 |  |
| 57801-72800 | 125 | 80.13 |  | 31 | 19.87 |  |
| >=72801 | 155 | 75.24 |  | 51 | 24.76 |  |
| Occupation |  |  |  |  |  | <.01** |
| unemployed | 955 | 67.54 |  | 459 | 32.46 |  |
| private employee and government | 3,831 | 76.09 |  | 1,204 | 23.91 |  |
| labor union member | 815 | 66.05 |  | 419 | 33.95 |  |
| farmer and fisherman | 257 | 62.38 |  | 155 | 37.62 |  |
| soldier | 20 | 74.07 |  | 7 | 25.93 |  |
| social | 83 | 63.85 |  | 47 | 36.15 |  |
| veteran | 1,083 | 65.72 |  | 565 | 34.28 |  |
| Level of urbanization |  |  |  |  |  | 0.49 |
| highly urbanized area | 2,471 | 70.88 |  | 1,015 | 29.12 |  |
| moderately urbanized area | 2,297 | 70.55 |  | 959 | 29.45 |  |
| emerging area | 1,152 | 72.54 |  | 436 | 27.46 |  |
| general area | 785 | 72.69 |  | 295 | 27.31 |  |
| aging area | 87 | 65.91 |  | 45 | 34.09 |  |
| agricultural area | 125 | 69.44 |  | 55 | 30.56 |  |
| remote area | 127 | 71.35 |  | 51 | 28.65 |  |
| Location |  |  |  |  |  | <.01** |
| taipei division | 2,985 | 71.17 |  | 1,209 | 28.83 |  |
| northern division | 1,187 | 75.61 |  | 383 | 24.39 |  |
| central division | 1,100 | 70.74 |  | 455 | 29.26 |  |
| southern division | 715 | 68.42 |  | 330 | 31.58 |  |
| kaoping division | 894 | 68.88 |  | 404 | 31.12 |  |
| eastern division | 163 | 68.49 |  | 75 | 31.51 |  |

| Table S6. Chi-square test of matched patient samples for obesity | | |  |  |  |  |
| --- | --- | --- | --- | --- | --- | --- |
| **Variable** | **Obesity** | | | | | **P-value** |
|  | **absent** | |  | **present** | |  |
|  | **N** | **%** |  | **N** | **%** |  |
| MD |  |  |  |  |  | <.01** |
| absent | 84,636 | 98.96 |  | 892 | 1.04 |  |
| present | 83,904 | 98.1 |  | 1,624 | 1.9 |  |
| **More than two MD comorbidities** |  |  |  |  |  | <.01** |
| absent | 49,192 | 98.54 |  | 727 | 1.46 |  |
| present | 39,678 | 97.75 |  | 912 | 2.25 |  |
| MD-type |  |  |  |  |  | <.01** |
| absent | 84,636 | 98.96 |  | 892 | 1.04 |  |
| affective disorder | 7,991 | 97.5 |  | 205 | 2.5 |  |
| anxiety disorder | 38,706 | 97.99 |  | 792 | 2.01 |  |
| substance use disorder | 8,176 | 98.86 |  | 94 | 1.14 |  |
| schizophrenia | 1,354 | 96.3 |  | 52 | 3.7 |  |
| other MD | 27,677 | 98.29 |  | 481 | 1.71 |  |
| Gender |  |  |  |  |  | <.01** |
| female | 83,462 | 98.12 |  | 1,603 | 1.88 |  |
| male | 85,078 | 98.94 |  | 913 | 1.06 |  |
| Age (years) |  |  |  |  |  | <.01** |
| 20-24 | 6,471 | 98.78 |  | 80 | 1.22 |  |
| 25-34 | 24,791 | 98.4 |  | 403 | 1.6 |  |
| 35-44 | 34,322 | 98.34 |  | 580 | 1.66 |  |
| 45-54 | 35,459 | 98.41 |  | 574 | 1.59 |  |
| 55-64 | 31,182 | 98.38 |  | 514 | 1.62 |  |
| >=65 | 36,315 | 99 |  | 365 | 1 |  |
| Income (NTD) |  |  |  |  |  | <.01** |
| <=20008 | 116,524 | 98.68 |  | 1,564 | 1.32 |  |
| 20009-22800 | 15,436 | 98.06 |  | 305 | 1.94 |  |
| 22801-28800 | 8,883 | 98.2 |  | 163 | 1.8 |  |
| 28801-36300 | 8,729 | 98.28 |  | 153 | 1.72 |  |
| 36301-45800 | 9,429 | 98.23 |  | 170 | 1.77 |  |
| 45801-57800 | 3,789 | 98.36 |  | 63 | 1.64 |  |
| 57801-72800 | 3,047 | 98.23 |  | 55 | 1.77 |  |
| >=72801 | 2,703 | 98.43 |  | 43 | 1.57 |  |
| Occupation |  |  |  |  |  | <.01** |
| unemployed | 16,700 | 98.44 |  | 265 | 1.56 |  |
| private employee and government | 76,377 | 98.57 |  | 1,109 | 1.43 |  |
| labor union member | 23,396 | 98.3 |  | 405 | 1.7 |  |
| farmer and fisherman | 17,871 | 98.79 |  | 218 | 1.21 |  |
| soldier |  |  |  |  |  |  |
| social | 1,960 | 97.37 |  | 53 | 2.63 |  |
| veteran | 32,236 | 98.58 |  | 466 | 1.42 |  |
| **Comorbidity (CCI)** |  |  |  |  |  | <.01** |
| 0 | 124,188 | 98.69 |  | 1,650 | 1.31 |  |
| >=1 | 44,352 | 98.08 |  | 866 | 1.92 |  |
| Taking drugs with obesity side effects |  |  |  |  |  | <.01** |
| no | 167,547 | 98.54 |  | 2,474 | 1.46 |  |
| yes | 993 | 95.94 |  | 42 | 4.06 |  |
| Level of urbanization |  |  |  |  |  | <.01** |
| highly urbanized area | 52,005 | 98.4 |  | 847 | 1.6 |  |
| moderately urbanized area | 50,927 | 98.58 |  | 731 | 1.42 |  |
| emerging area | 27,622 | 98.6 |  | 392 | 1.4 |  |
| general area | 22,780 | 98.59 |  | 326 | 1.41 |  |
| aging area | 4,060 | 99.02 |  | 40 | 0.98 |  |
| agricultural area | 5,924 | 98.19 |  | 109 | 1.81 |  |
| remote area | 5,222 | 98.66 |  | 71 | 1.34 |  |
| Location |  |  |  |  |  | <.01** |
| taipei division | 62,178 | 98.45 |  | 982 | 1.55 |  |
| northern division | 24,505 | 98.65 |  | 335 | 1.35 |  |
| central division | 30,255 | 98.73 |  | 388 | 1.27 |  |
| southern division | 22,201 | 98.56 |  | 325 | 1.44 |  |
| kaoping division | 25,501 | 98.52 |  | 383 | 1.48 |  |
| eastern division | 3,900 | 97.43 |  | 103 | 2.57 |  |
